# Comprehensive protein synthesis inhibition impairs natural and artificial memory recall

**DOI:** 10.1186/s13041-026-01305-2

**Published:** 2026-04-29

**Authors:** Yeonjun Kim, Ilgang Hong, Bong-Kiun Kaang

**Affiliations:** 1 Center for Memory and Glioscience, Institute for Basic Science (IBS), Daejeon, South Korea; 2 Interdisciplinary Program in Neuroscience, Seoul National University, Seoul, South Korea; 3 Department of Biological Sciences, College of Natural Sciences, Seoul National University, Seoul, South Korea; 4 Department of Biological Sciences, Korea Advanced Institute of Science and Technology, Daejeon, South Korea

**Keywords:** Protein synthesis inhibitor, Engram, Artificial memory recall, Anisomycin, Cycloheximide

## Abstract

Protein synthesis is critical for long-lasting memory formation and synaptic plasticity. However, whether protein synthesis is required for artificially evoked memory retrieval remains debated. To address this, we used a translation-inhibitor cocktail (CKT; anisomycin and cycloheximide) to achieve a more comprehensive blockade of protein synthesis compared to more commonly used anisomycin alone (ANI). When administered immediately after memory acquisition, ANI impaired natural recall while still showing preserved artificial recall. In contrast, CKT treatment showed significant impairment in both natural and artificial recall. Together, these results provide additional evidence that de novo protein synthesis is required for both natural and artificial memory recall.

**Supplementary Information:**

The online version contains supplementary material available at 10.1186/s13041-026-01305-2.

## Main text

Protein synthesis is required for enduring forms of memory and synaptic plasticity in the nervous systems of both vertebrates and invertebrates [1, 2]. Applying a protein synthesis inhibitor (PSI) during memory acquisition blocks the late phase of long-term potentiation [3] which ultimately impair natural recall [4]. However, whether protein synthesis is required for artificial memory recall remains elusive. Artificial memory recall can be achieved by directly activating engram neurons through DREADDs (designer receptors exclusively activated by designer drugs) [5] or excitatory opsins [6]. While some studies report that artificial recall is largely unaffected by protein synthesis inhibition [4, 7], others suggest that artificial recall becomes impaired when protein synthesis is inhibited [8, 9]. We recently demonstrated that prolonged protein synthesis inhibition over a 6-h window abolishes artificial recall, supporting a critical role for protein synthesis even in artificially evoked memory retrieval [10]. However, it remains unclear whether more comprehensive blockade of protein synthesis, using a cocktail of PSIs [11], also impairs artificial memory retrieval.

In this study, we used anisomycin in combination with cycloheximide as a translation-inhibitor cocktail. Anisomycin inhibits translation by targeting the peptidyl transferase center of the 60S ribosomal subunit, thereby suppressing peptidyl transferase activity [12]. Cycloheximide, on the other hand, binds to the E-site of the 60S subunit, thereby interfering with mRNA translocation [13].

To first confirm that protein synthesis inhibition disrupts natural memory recall, engram reactivation analysis was performed. ArcTRAP mice crossed with floxed tdTomato mice were used to label activated neurons with tdTomato driven by the promoter of the immediate early gene Arc. These mice underwent contextual fear conditioning (CFC), followed by 4-hydroxytamoxifen (4-OHT) injection (Supplementary Fig. 1). Mice then received saline (SAL), 150 mg/kg of anisomycin (ANI), or a cocktail of 150 mg/kg of anisomycin and 30 mg/kg of cycloheximide (CKT). The dosage of each of the drugs were chosen as they have previously been used to effectively inhibit learning and memory in mice [14, 15]. Two days later, mice underwent a retrieval session to assess natural recall. Both the ANI and CKT groups showed significantly impaired natural recall (Supplementary Fig. 1B). Ninety minutes after retrieval, mice were perfused for Fos immunohistochemistry. Notably, the CKT group exhibited a significantly lower engram reactivation ratio in the basal amygdala (BA) compared to the ANI group (Supplementary Fig. H). Together, these data indicate that comprehensive protein synthesis inhibition via a translation-inhibitor cocktail impairs natural recall, with concomitant reduction in engram reactivation.

**Fig. 1:**
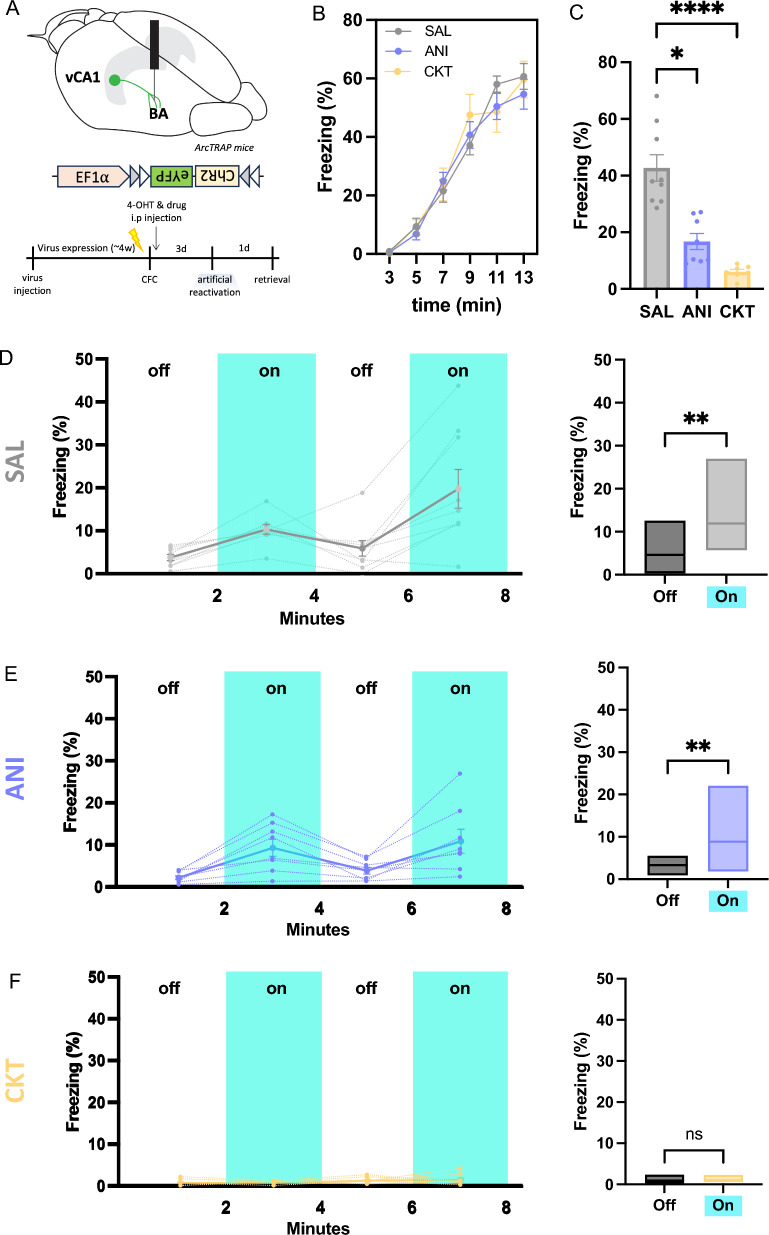
Artificial memory recall is disabled when protein synthesis is blocked during memory acquisition with a combination of PSIs. **A** Schematic overview of the experiment. **B** Freezing level during memory acquisition. Data are presented as mean ± SEM. **C** Freezing level during contextual fear memory retrieval. SAL group, N = 9; ANI group, N = 8; CKT group, N = 7. Kruskal-Wallis test followed by Dunn’s multiple comparison test. *P = 0.0380; ****P < 0.0003. Data are presented as mean ± SEM. **D**–**F** Optogenetic activation of vCA1 engram axon terminals in BA of mice treated with different combination of PSI during memory acquisition. Mice expressing ChR2 in vCA1 engram cells show greater freezing during light-on epochs in the SAL group (**C**) or the ANI group (**D**) while absence of freezing behavior was shown in the CKT group (**E**). Wilcoxon test. SAL group, **P = 0.0039; ANI group, **P = 0.0078. Data are presented as mean ± SEM (left) or median (right).

To test whether protein synthesis inhibition affects artificial memory recall, we expressed the excitatory opsin ChR2 in ventral CA1 (vCA1) engram neurons of ArcTRAP mice (Fig. 1A). The vCA1 to BA circuit has been shown to be necessary and sufficient for memory retrieval [10, 16]. Therefore, an optical fiber was implanted above the BA to stimulate BA-projecting vCA1 engram axon terminals. Mice underwent CFC and were immediately injected with the corresponding drugs and 4-OHT. All three groups of mice did not show a difference in memory acquisition (Fig. 1B). ANI showed significant impairment in natural recall, consistent with previous studies [4, 7, 10], and CKT produced an even more robust impairment (Fig. 1C).

Artificial reactivation was performed in a novel context by optogenetically stimulating BA-projecting vCA1 engram axon terminals (Fig. 1D–F). All groups showed minimal freezing in the absence of blue light stimulation. In contrast, blue light stimulation induced significantly higher freezing in the SAL group, indicating that reactivating BA-projecting vCA1 engram axon terminals is sufficient in eliciting memory recall (Fig. 1D). Similarly, the ANI group also exhibited significantly higher freezing upon blue light stimulation, despite impaired natural recall, consistent with previous studies [4, 7, 10] (Fig. 1E). Surprisingly, the CKT group did not show significant freezing in response to the same optogenetic stimulation protocol (Fig. 1F). These data suggest that de novo protein synthesis is required not only for natural recall but also for artificial memory recall.

In this study, we found that a more comprehensive blockade of protein synthesis impairs both natural and artificial recall. Although, the mechanism by which CKT disrupts artificial recall was not directly investigated in this study, one plausible explanation is that CKT more effectively suppresses learning-induced strengthening of engram synapses. Strengthening of engram synapses (synapses between engram cells) has been shown to correlate with memory formation [10, 17]. It has been recently shown that a single anisomycin injection prevents engram synapse enlargement while largely unaffecting engram synapse formation [10]. Therefore, the behavioral phenotype observed in the ANI group (impaired natural recall with preserved artificial recall) may be due to selective inhibition of the engram synapse enlargement (Fig. 1E). In contrast, prolonged protein synthesis inhibition spanning a 6-h window has been shown to prevent both engram synapse formation and enlargement and to impair both natural and artificial recall [10]. This behavioral phenotype resembles that of the CKT group in this study (Fig. 1F). Thus, the combined deficits in natural and artificial recall following CKT treatment may similarly arise from a more complete blockade of learning-induced engram synapse formation and enlargement.

## Materials and methods

### Animals

All experiments were conducted in accordance with the regulations and guidelines of the Institutional Animal Care and Use Committees (IACUC) of Seoul National University (SNUIBC-220508–3) and Institute for Basic Science (IBS-2023–020).

Heterozygous Arc-CreERT2 (Jackson Laboratory Stock # 022357) mice, obtained by crossing wild-type C57BL6/J and Arc-CreERT2 (+ / −) mice, were utilized. Mice were housed in standard laboratory cages with disposable bedding on a standard light cycle (09 h–21 h). Mice had access to food and water ad libitum and were socially housed in numbers of three to five littermates until surgery. Male mice were used for the experiments. Behavioral experiments were performed during the light cycle.

### Stereotaxic surgery procedure

Mice were anaesthetized by intraperitoneal injection of 25 mg·kg^− 1^ ketamine/xylazine and positioned on a stereotaxic apparatus (Stoelting Co.). Ophthalmic ointment was applied to the eyes as a preventive measure against dryness. Bilateral craniotomies were performed using a 0.5 mm diameter drill and the viruses were injected using a micro-needle attached to a 10 mL Hamilton micro syringe (Hamilton; cat# 701LT). A syringe driver (WPI, cat# SP3101-PLUS) was used to maintain the speed of the injection at 0.125 ul·min^− 1^. The needle was slowly lowered to the target site and remained for two minutes before beginning the injection. After injection, the needle stayed for 10 min before it was slowly withdrawn. A custom implant containing two optic fibers (Newdoon; 0.37 NA 1.25 mm core diameter) was lowered above the injection site at the following coordinate relative to bregma (mm): AP: -1.5 ML: ± 3.0 DV: − 4.0 for BA. A jewelry screw was screwed into the skull near the bregma. A layer of adhesive cement (C&B Metabond) was applied followed by dental cement (A-M Systems; Teets cold cure) to secure the optic implant.

AAV9-EF1α-DIO-ChR2-EYFP was acquired from Institute for Basic Science Recombinant Virus Packaging Facility. The recombinant AAV vectors were injected with viral titers of 6.0 × 10^12^ GC·ml^− 1^.

### Contextual fear conditioning

Prior to conditioning, each mouse was housed individually for eight days and was subjected to daily handling sessions lasting five minutes for seven consecutive days. Mice were transferred from the vivarium to a holding room adjacent to the testing area at least 30 min prior to the start of the experiment. Furthermore, each mouse was transported individually to the conditioning room.

The conditioning chamber measuring 7″W×7″D×12″H (Coulbourn Instruments; model H10-11 M-TC) was used. The chamber featured two aluminum side walls and a ceiling with an acrylic door. It was situated within a larger sound-attenuating enclosure. Throughout the experimental procedures, a white overhead light continuously illuminated the conditioning chamber. The chamber itself was equipped with a straight stainless-steel rod floor which was cleaned using a 70% ethanol solution and distilled water between each use.

For CFC, mice were exposed to the context for 180 s, after which five-foot shocks (0.5 mA, 2 s) were delivered where each foot shock lasted two seconds with an interval of two minutes. Following the conditioning session, mice were returned to their respective home cages. During the retrieval test, the percentage of time spent freezing was calculated and averaged across the entire three-minute retrieval session.

Anisomycin (Tocris, cat# 1290) and cycloheximide (Sigma Aldrich, cat# 239,763-M) were stored at − 20 °C up to a year, and the powder was dissolved with saline on the day of use. Mice were either injected with saline, anisomycin, or anisomycin + cycloheximide right after conditioning. 4-OHT (Sigma Aldrich, cat# H6278) was injected intraperitoneally for 50 mg·kg^− 1^ 10 min after the first injection.

### Immunohistochemistry

Ninety minutes after retrieval, mice were deeply anesthetized using ketamine/xylazine (15 mg·kg^− 1^) followed by transcranial perfusion with 15 ml of PBS and 15 ml of 4% PFA. Brains were removed, post-fixed overnight at 4 °C in 4% PFA and dehydrated in a 30% sucrose solution in PBS at 4 °C for two days. The brain was rinsed with PBS and subsequently frozen at − 80 °C for a minimum of one hour. Coronal sectioning was performed using a cryostat (Leica) with a thickness of 30 μm. The sections were rinsed with 1 mL of PBS for 5 min each time at room temperature (RT), 120 rpm. Then 500 μL of a blocking solution (5% normal goat serum, 0.3% triton X-100 in PBS) was applied to each sample for 1 h RT, 80 rpm. The primary c-Fos antibody (SySy, cat# 226 003) was applied at a concentration of 1:1,000 for 24 h on a shaker at 4 °C. Sections were washed three times with PBS for 5 min each time at RT, 120 rpm. A secondary antibody (Alexa-488; 1:500) was applied at a concentration of 1:500 for 2 h at 80 rpm. Finally, sections were washed with 1 mL of PBS three times for 5 min each time at 120 rpm, RT. The second washing step included DAPI staining at a concentration of 1:1,000 for 5 min at 80 rpm, RT. Cell counting was manually performed using Imaris (Bitplane) by an experimenter who was blinded to the conditions.

### Optogenetics

Mice were habituated to optical cables for at least 30 min before the experiment. Mice were habituated in their home cage with an optical cable connected. From 30 to 60 min of habituation, mice were placed in the acrylic chamber. Mice were allowed to freely move within the acrylic chamber and freezing behavior was recorded for 8 min, with two minutes of two consecutive lights ‘off’ and ‘on’ sessions. During the light ‘on’ session, mice received two minutes of 20 Hz pulses of 473 nm laser through an optical cannula above the BA region (20 mW/mm^2^, 2 ms pulses).

### Statistical analysis

Data analysis was performed on Prism (GraphPad Software). The α value was set at 0.05 for all analyses.

## Supplementary Information

Supplementary Material 1.

## Data Availability

The datasets used and/or analyzed during the current study are available from the corresponding author upon reasonable request.

## References

[1] Kang H, Schuman EM. A requirement for local protein synthesis in neurotrophin-induced hippocampal synaptic plasticity. Science. 1996;273:1402–6. 8703078 10.1126/science.273.5280.1402

[2] Lee SH, et al. Nuclear translocation of CAM-associated protein activates transcription for long-term facilitation in aplysia. Cell. 2007;129:801–12. 17512412 10.1016/j.cell.2007.03.041

[3] Schafe GE, LeDoux JE. Memory consolidation of auditory Pavlovian fear conditioning requires protein synthesis and protein kinase A in the amygdala. J Neurosci. 2000. 10.1523/jneurosci.20-18-j0003.2000. 10974093 10.1523/JNEUROSCI.20-18-j0003.2000 PMC6772816

[4] Ryan TJ, Roy DS, Pignatelli M, Arons A, Tonegawa S. Engram cells retain memory under retrograde amnesia. Science. 2015;348:1007–13. 26023136 10.1126/science.aaa5542 PMC5583719

[5] Roth BL. DREADDs for neuroscientists. Neuron. 2016;89:683–94. 26889809 10.1016/j.neuron.2016.01.040 PMC4759656

[6] Liu X, et al. Optogenetic stimulation of a hippocampal engram activates fear memory recall. Nature. 2012;484:381–5. 22441246 10.1038/nature11028 PMC3331914

[7] Roy DS, Muralidhar S, Smith LM, Tonegawa S. Silent memory engrams as the basis for retrograde amnesia. Proc Natl Acad Sci U S A. 2017;114:E9972–9. 29078397 10.1073/pnas.1714248114 PMC5699085

[8] Abdou K, et al. Synapse-specific representation of the identity of overlapping memory engrams. Science. 2018;360:1227–31. 29903972 10.1126/science.aat3810

[9] Kim CH, et al. Growth hormone is required for hippocampal engram cell maturation. Sci Adv. 2025;11(51):eaec7836. 41417890 10.1126/sciadv.aec7836 PMC12716386

[10] Hong I, et al. Protein synthesis blockade prevents fear memory reactivation via inhibition of engram synapse strengthening. Proc Natl Acad Sci U S A. 2026;123:e2510016123. 41525485 10.1073/pnas.2510016123 PMC12818406

[11] Cleynen A, Ravindran A, Shirokikh N. FracFixR: a compositional statistical framework for absolute proportion estimation between fractions in RNA sequencing data. bioRxiv. 2025. 10.1101/2025.07.29.667459. 10.1093/bioinformatics/btaf615 PMC12866640 41264734

[12] Barbacid M, Vazquez D. [3H]anisomycin binding to eukaryotic ribosomes. J Mol Biol. 1974;84:603–23. 4601392 10.1016/0022-2836(74)90119-3

[13] Garreau De Loubresse N, et al. Structural basis for the inhibition of the eukaryotic ribosome. Nat. 2014;513(7519):517–22. 10.1038/nature13737 25209664

[14] Leccese AP, Quinton EE. Attenuation of cycloheximide-induced amnesia in mice with strychnine sulfate. Behav Neurosci. 1983;97:323–6. 6682670 10.1037//0735-7044.97.2.323

[15] Kinsky NR, et al. Erasable hippocampal neural signatures predict memory discrimination. Cell Rep. 2025;44:115391. 40057951 10.1016/j.celrep.2025.115391 PMC12517107

[16] Kim WB, Cho JH. Encoding of contextual fear memory in hippocampal–amygdala circuit. Nat Commun. 2020;11:1–22. 32170133 10.1038/s41467-020-15121-2 PMC7069961

[17] Choi JH, et al. Interregional synaptic maps among engram cells underlie memory formation. Science. 2018;360:430–5. 29700265 10.1126/science.aas9204

