# Supplementary material for: Comprehensive protein synthesis inhibition impairs natural and artificial memory recall
Source: Mol Brain. 2026 Apr 29;19:46. doi: 10.1186/s13041-026-01305-2 (PMC13270719; doi:10.1186/s13041-026-01305-2)
Supplement: Supplementary file 1 — Supplementary Material 1. [file 13041_2026_1305_MOESM1_ESM.pdf]

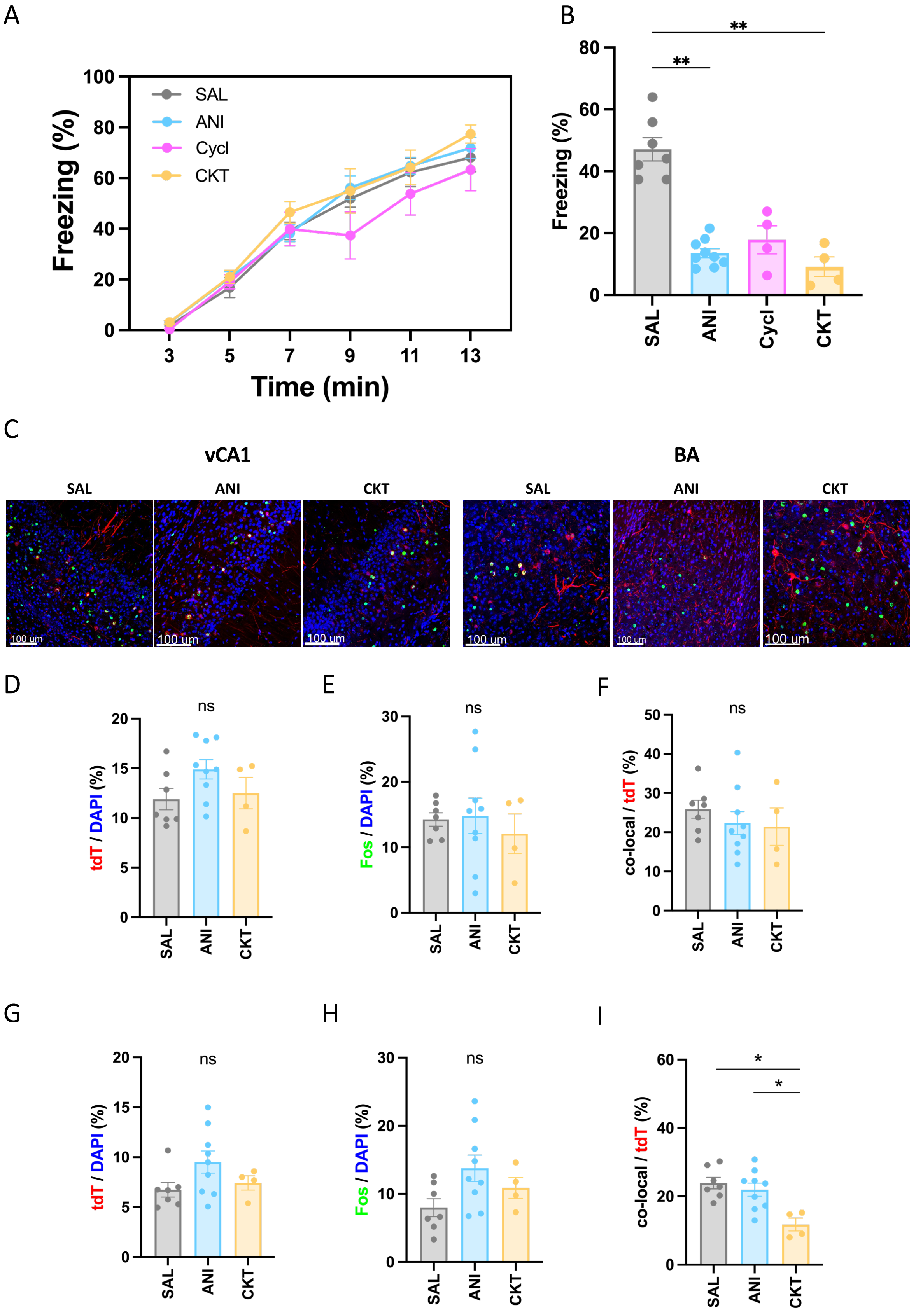

A
Freezing (%)
Time (min)
SAL
ANI
Cycl
CKT
B
Freezing (%)
**
**
SAL
ANI
Cycl
CKT
C
vCA1
BA
SAL
ANI
CKT
SAL
ANI
CKT
100 um
D
tdT / DAPI (%)
ns
E
Fos / DAPI (%)
ns
F
co-local / tdT (%)
ns
G
tdT / DAPI (%)
ns
H
Fos / DAPI (%)
ns
I
co-local / tdT (%)
*
*

Supplementary Fig. 1: Protein synthesis inhibition impairs natural memory recall and reduces engram reactivation in the BA.
(**A**) Acquisition plot for SAL, ANI, Cycl, and CKT group during CFC. SAL group, N = 7; ANI group, N = 9; Cycl group, N = 4; CKT group, N = 4. Cycl group is treated only with 30 mg/kg of cycloheximide. Data are presented as mean ± SEM.
(**B**) Freezing level during memory recall. Kruskal-Wallis test followed by Dunn's multiple comparison test. SAL vs. ANI, **P = 0.0057; SAL vs. CKT, **P = 0.0035. Data are presented as mean ± SEM.
(**C**) Representative images for Fos immunohistochemistry for vCA1 and BA.
(**D-F**) Immunohistochemistry data of vCA1. Kruskal-Wallis test followed by Dunn's multiple comparison test. Data are presented as mean ± SEM.
(**G-I**) Immunohistochemistry data of BA. Kruskal-Wallis test followed by Dunn's multiple comparison test. SAL vs. CKT, *P = 0.0148; ANI vs. CKT, *P = 0.0483. Data are presented as mean ± SEM.
